# Monocyte and Lymphocyte Count, and Lymphocyte/Monocyte Ratio as Prognostic Factors at the Time of First Relapse in Canine Diffuse Large B-Cell Lymphoma Patients Receiving Chemotherapy

**DOI:** 10.3390/ani16010009

**Published:** 2025-12-19

**Authors:** Sara Cermeno, Alenka Lavra Zajc, Tim Sparks, Carlota Carvalho Molina, Adam Swallow

**Affiliations:** 1 Anicura Canis i Felis, c. Bogatell 71, 08930 Sant Adrià de Besòs, Barcelona, Spain; 2 Northwest Veterinary Specialists, Delamere House, Ashville Point, Sutton Weaver, Runcorn WA7 3FW, UK; 3 Waltham Petcare Science Institute, Freeby Lane, Waltham on the Wolds, Melton Mowbray, Leicestershire LE14 4RT, UK; 4 North Shore Veterinary Specialist and Emergency Centre, 63 Herbert St, Artarmon, NSW 2064, Australia

**Keywords:** DLBCL—diffuse large B-cell lymphoma, LMR—lymphocyte-to-monocyte ratio, OS—overall survival, OS_r_—overall survival from relapse, DFI—disease free interval, MST—median survival time, LSS—lymphoma-specific survival, PFS—progression free survival

## Abstract

Background: Canine lymphoma comprises the majority of haematopoietic malignancies in veterinary clinical practice. Several prognostic factors have been studied and, more recently, there has been an increased interest in the role of the lymphocyte-to-monocyte ratio (LMR) for its prognostic value. To date, the prognostic value of absolute monocyte and lymphocyte counts as well as LMR at the time of relapse in dogs with diffuse large B-cell lymphoma has not been evaluated. The purpose of the present study was to investigate whether the absolute monocyte, lymphocyte or LMR at relapse can predict clinical outcomes for relapsed diffuse large B-cell lymphoma dogs treated with chemotherapy. Additionally, the parameters were evaluated for their prognostic value at the time of diagnosis and throughout different timepoints during the course of their first-line chemotherapy treatment. Materials and Methods: We retrospectively analysed data from 50 dogs with relapsed diffuse large B-cell lymphoma, treated with a CEOP-based first-line chemotherapy protocol. Lymphocyte and monocyte count and LMR were evaluated at different timepoints: at diagnosis, during chemotherapy and at the time of relapse. Overall survival time (OS) and disease-free interval (DFI), as well as overall survival time from relapse (OS_r_), were measured. Friedman nonparametric ANOVA was used to compare blood cell counts at different timepoints. Spearman rank correlation was used to test for association between blood cell count at various timepoints with the duration of remission and survival time. Results: Monocyte and lymphocyte counts and LMR at the time of first relapse were not found to be adverse prognostic factors for OS_r_ in this population of dogs. Monocyte and lymphocyte counts differed significantly between different timepoints during the chemotherapy protocol. Conclusions: Absolute monocyte and lymphocyte counts and LMR at the time of relapse were not found to be prognostic indicators of OS_r_ in this population of dogs with multicentric lymphoma. Additional studies evaluating absolute monocyte and lymphocyte counts during chemotherapy treatment and following completion of chemotherapy in larger population of dogs are needed to assess whether these counts have clinical utility in detecting disease progression.

## Simple Summary

Canine lymphomas account for the majority of haematopoietic tumours in veterinary clinical practice. Several prognostic factors have been evaluated in dogs with diffuse large B-cell lymphoma (DLBCL) and, more recently, the role of leukocytes has been of increased interest. The main objective of this study was to assess the prognostic value of absolute monocyte and lymphocyte count, as well as lymphocyte-to-monocyte ratio (LMR), at the time of first relapse in a population of dogs with diffuse large B-cell lymphoma treated with CEOP-based chemotherapy. Additionally, absolute monocyte and lymphocyte count, as well as LMR, were evaluated for their prognostic value at the time of diagnosis and throughout different timepoints during the course of the chemotherapy treatment. Monocyte counts, lymphocyte counts, and their ratio were not found to be significant predictors of overall survival at relapse or at the time of diagnosis in this population of dogs. Furthermore, our findings suggest that monitoring of the absolute lymphocyte count throughout chemotherapy treatment and during follow-up in these dogs may be of clinical help to identify disease progression. Larger, controlled prospective studies evaluating blood cell counts throughout the course of chemotherapy and at relapse are needed to evaluate these findings further.

## 1. Introduction

Canine lymphoma comprises the majority of haematopoietic malignancies seen in veterinary clinical practice [1,2]. Multicentric lymphoma is the most common presentation of lymphoma in dogs, characterised by the presence of multiple lymph node enlargement and diffuse large B-cell lymphoma is the most common subtype [3,4,5]. The use of multidrug cytotoxic protocols is the standard of care for canine multicentric lymphomas. The multidrug combination, including vincristine, cyclophosphamide, doxorubicin and oral prednisolone, known as the CHOP protocol, is the current standard therapy for diffuse large B-cell canine multicentric lymphoma. This is associated with good initial responses and a reported overall response rate (ORR) of 96.3–100%. Progression-free survival (PFS) times are reported at 233–252 days and overall median survival times (MST) at 316–341 days, with 20–25% of dogs being alive at 2 years [6,7,8]. Historically, the CHOP protocol was used irrespective of immunophenotype; however, there is increasing evidence that alkylator-rich multidrug protocols provide improved ORR, PFS and MST in non-indolent T-cell lymphomas [9,10,11,12] in comparison to CHOP protocols, with reported ORR of 94–97%, PFS of 176–431 days and MST of 323–507 days [10,11]. Furthermore, epirubicin is sometimes used in place of doxorubicin as part of a multi-agent chemotherapy protocol in some oncology centres, due to reported reduced cardiotoxicity and equipotent activity [13], although it may result in a higher incidence of adverse gastrointestinal effects [14]. Unfortunately, the majority of dogs with lymphoma eventually experience relapse of disease, with progressive disease and ultimately, death, despite treatment [8].

Several prognostic factors have been studied, including clinical stage and substage [15,16], immunophenotype [16,17], grade [16] and anatomical form [18]. More recently, there has been an increased interest in the role of monocyte and lymphocyte counts for their predictive value in different malignancies in humans and dogs [19,20,21,22,23,24,25,26,27,28,29,30,31,32,33,34,35].

In humans, the presence of high monocyte and low lymphocyte counts at the time of diagnosis have been associated with a poorer prognosis in patients with different types of cancers [23,29], and absolute peripheral monocyte count at diagnosis has been found to be a predictor for central nervous system relapse in people with diffuse large B-cell lymphoma [36]. Studies in dogs with lymphoma have shown that monocytosis at the time of diagnosis is associated with decreased disease-free interval (DFI) [19], and that the absolute monocyte count is independently associated with time to progression (TTP) and lymphoma-specific survival (LSS) [21]. No predictive value for the absolute lymphocyte count has been found in dogs with lymphoma.

Moreover, the prognostic value of the lymphocyte-to-monocyte ratio (LMR) at the time of diagnosis has also been reported in people with diffuse large B-cell lymphoma [25,26,27]. Similarly in dogs, LMR at diagnosis ≤ 1.2 was found to have significantly shorter TTP and lymphoma-specific survival as an independent prognostic indicator in dogs with diffuse large B-cell lymphoma treated with chemoimmunotherapy (combination of CHOP protocol with APAVAC immunotherapy) [21]. Other studies also found that LMR at diagnosis above 1.43 was an independent predictive factor of longer OS [8], and that an LMR cut off value of 0.7 corresponded to a maximum sensitivity and specificity for predicting OS in canine high-grade lymphoma cases [28]. Additionally, a recent publication found that low LMR had significant reductions in both TTP and OS in cats [37].

More recently, the prognostic value of LMR at the time of the first relapse was evaluated in people with diffuse large B-cell lymphoma (DLBCL). Lower LMR was found to be an adverse prognostic factor for both OS and PFS in relapsed or primary refractory DLBCL [24,38]. Furthermore, studies in people also found that the absolute lymphocyte count at the time of first relapse may help to predict survival [39].

While prognostic value of absolute monocyte and lymphocyte count, and lymphocyte/monocyte ratio has been evaluated in dogs with DLBCL at the time of diagnosis, information on their prognostic value at the time of the first relapse is lacking.

### Objectives

Our main objective in this retrospective, observational study was to assess the prognostic value of absolute monocyte and lymphocyte count, as well as LMR, at the time of first relapse in our population of dogs with diffuse large B-cell lymphoma treated with CEOP-based chemotherapy.

We hypothesised that a lower LMR and an increase in the monocyte count and lower lymphocyte count at the time of first relapse would be an adverse prognostic factor for OSr in relapsed diffuse large B-cell lymphoma in dogs.

Additionally, absolute monocyte and lymphocyte count, as well as LMR, were evaluated for their prognostic value at the time of diagnosis and throughout different timepoints during the course of the chemotherapy treatment.

## 2. Materials and Methods

Medical records from January 2006 to January 2024 at Cave Veterinary Specialists (Wellington, UK) and Northwest Veterinary Specialists (Sutton Weaver, UK) were reviewed retrospectively for cases of multicentric high-grade canine lymphoma. Inclusion criteria included (1) dogs diagnosed with large B-cell multicentric lymphoma based on histology or cytology from a peripheral lymph node, reviewed by a board-certified clinical pathologist, (2) confirmed B-cell immunophenotype (on PARR (PCR for antigen receptor rearrangement), immunohistochemistry (IHC), immunocytochemistry (ICC) or flow cytometry (FC)), (3) dogs receiving a CEOP-based chemotherapy protocol as their first-line treatment, (4) dogs with haematology performed prior to each chemotherapy administration, (5) documented clinical remission and (6) reported clinical relapse, based on cytology and according to previously published response evaluation criteria for peripheral nodal lymphoma in dogs [18], with documented haematology records at the time of relapse, as well as the date when the relapse was reported. All dogs included were followed up with for at least 12 months from initiation of chemotherapy treatment (7), and further salvage chemotherapy or palliative treatments were recorded after first relapse (8). Finally, date of death or loss to follow-up for all dogs was included for statistical calculations (9).

**Figure 1:**
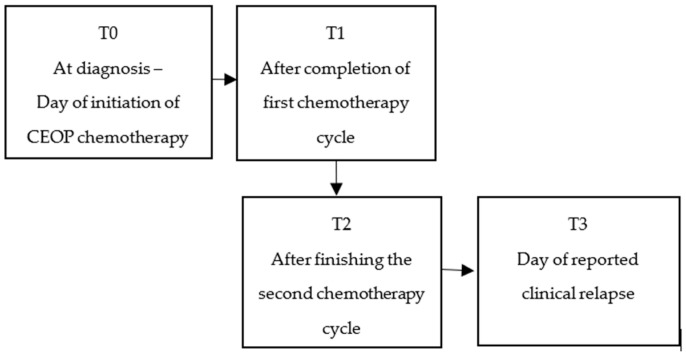
Timepoints throughout chemotherapy treatment when haematology was performed for monocyte and lymphocyte counts.

The monocyte and lymphocyte counts were recorded from haematology results for every dog, and the LMR was calculated in all dogs on the day of initiating chemotherapy treatment (T0); on the day of completion of the first cycle of chemotherapy (T1—recorded at week 5 in CEOP protocol and week 6 in modified-CEOP protocol), after completing the second cycle of treatment (T2—week 11 in CEOP protocol and week 9 in modified-CEOP protocol), and on the day of documented clinical relapse (T3). Clinical relapse was confirmed via fine-needle aspiration and cytology of the peripheral lymph nodes or according to previously published response evaluation criteria for peripheral nodal lymphoma in dogs [18] (see Figure 1).

### 2.1. Data Collection

Dogs were excluded if they did not meet the inclusion criteria, had incomplete data, if treatment was discontinued for reasons other than clinical relapse (owner’s decision, financial restrictions, or due to adverse effects of chemotherapy), if clinical relapse was early on during the protocol (before week 11) or if no relapse was recorded after completing CEOP protocol.

For each dog included in the study, the information extracted from the medical record included age at time of diagnosis, gender, neuter status, breed, physical examination findings and any significant blood test abnormalities at diagnosis and at the time of relapse. Cases where another underlying disease was mentioned at diagnosis, which could interfere with haematology results, were excluded. Haematology was performed on whole peripheral blood samples (extracted from either the jugular vein or cephalic veins) collected in 1 mL EDTA (Ethylenediaminetetra-acetic acid) tubes. An external reference laboratory (Veterinary Pathology Group—VPG, Unit 1b, Anning Dr, Babbage Way, Clyst Honiton, Exeter EX5 2FN, UK; or Antech Diagnostics Laboratory—Dick White Referrals, Station Farm, London Road, Six Mile Bottom, Cambridgeshire CB8 0UH, UK) were used to obtain haematology at diagnosis, during chemotherapy treatment and at relapse. This included blood smear examination by a laboratory technician, a board-certified clinical pathologist or a supervised resident. Lymphocyte count and monocyte count were derived from the standard complete blood count and LMR was calculated by dividing lymphocyte by the monocyte count.

Each cytology and histology sample at diagnosis and at relapse was reviewed by a board-certified specialist pathologist or a supervised resident from different external laboratories (Veterinary Pathology Group—VPG, Unit 1b, Anning Dr, Babbage Way, Clyst Honiton, Exeter EX5 2FN, UK; Idexx Laboratories, Grange House, Sandbeck Way, Wetherby, West Yorkshire LS22 7DN, UK; Antech Diagnostics Laboratory—Dick White Referrals, Station Farm, London Road, Six Mile Bottom, Cambridgeshire CB8 0UH, UK; or AXIOM Veterinary Laboratories Ltd., Manor House, Brunel Road, Newton Abbot, Devon, TQ12 4PB, UK), as per the discretion of the attending clinician. Immunophenotype information of PARR (PCR for antigen receptor rearrangement), immunohistochemistry (IHC), immunocytochemistry (ICC) and flow cytometry (FC) were also recorded.

When available, the results of the staging investigations were documented—including thoracic and abdominal imaging, the cytology of fine-needle aspirates of spleen, liver and abdominal/thoracic lymph nodes and bone marrow biopsy results.

All dogs included received either a CEOP (*n* = 33) or a modified-CEOP (*n* = 17) chemotherapy protocol as a first-line treatment. The CEOP chemotherapy protocol consisted of intravenous vincristine (0.5–0.75 mg/m^2^), oral cyclophosphamide (200–250 mg/m^2^), intravenous epirubicin (30 mg/m^2^) and oral prednisolone tapered over 4 weeks. This drug combination was repeated for 4 cycles as detailed in Table 1, and complete protocol consisted of 25 weeks of treatment. Seventeen dogs received a modified-CEOP protocol, where intravenous vincristine was administered at week 1, oral cyclophosphamide at week 2, and intravenous epirubicin at week 3, as per doses above. This drug combination was repeated for 4 cycles as detailed in Table 2 and complete protocol consisted of 16 weeks of therapy.

The administration, or lack thereof, of prednisolone alongside cytotoxic drugs was also recorded. The dogs receiving L-asparginase therapy within the first week of initiating naïve chemotherapy treatment were included.

Response to treatment was evaluated at each treatment session and then every 1–2 months at the referring practice or at the referral hospital after finishing the protocol according to previously published response evaluation criteria for peripheral nodal lymphoma in dogs [18]. After the first relapse was reported, the records for each dog were followed and the first salvage treatment used was recorded.

Follow-up time was calculated for each patient and defined as the time between the initiation of the chemotherapy treatment and the day of recorded death or to last follow-up. Disease-free interval (DFI) was defined as the time from first documented clinical remission to time of clinical relapse. Lymphoma-specific survival (LSS) was defined as the time from the diagnosis until the day of recorded death due to lymphoma. Dogs were censored for LSS if lost to follow-up, with the last day of contact recorded, or if they remained alive at the end of the study period. Overall survival time (OS) was defined as the time from diagnosis until death. Dogs were censored for OS if they were lost to follow-up or still alive at the end of the study period, with the last day of contact recorded. Overall survival time from relapse (OS_r_) was defined as the time from the first relapse until death. Dogs were censored for OS_r_ if lost to follow-up, recording the last day of contact or if they were still alive at the end of the study period.

### 2.2. Statistical Analysis

Nonparametric tests have been used throughout because of the highly skewed nature of the data. Friedman nonparametric ANOVA adjusted for ties was used to compare timepoints (T0, T1, T2 and T3) in monocyte count, lymphocyte count and LMR while considering dogs as a blocking factor. Statistically significant results were followed by pairwise comparisons between timepoints based on Benjamini–Hochberg adjustment using the R package PMCMRplus (version 1.9.12.).

Spearman rank correlation was used to test for association between monocyte count, lymphocyte count and LMR at various timepoints with days to start of remission (T0 data only), disease-free interval, overall survival time and overall survival from relapse. No adjustment to significance has been made for multiple correlation tests. Comparisons with survival time, including censored data, were made using a Kaplan–Meier plot and a log-rank test. Mann–Whitney tests adjusted for ties were used to compare changes in monocyte count, lymphocyte count and LMR between T1 and T2 between dogs given or not given prednisolone.

Statistical significance was taken as *p* < 0.05. Analysis was performed in Minitab 21 and R 4.3.3.

## 3. Results

### 3.1. Study Population

Two hundred thirty-three (233) cases were identified for initial review. After reviewing each patient and applying the inclusion criteria, 183 cases were excluded, and 50 dogs were finally included in the study (Figure S1, Supplementary Material).

Median (range) age at diagnosis was 8 (2–14) years, 20 dogs (40%) were spayed females, 3 dogs (6%) were intact males and 27 dogs (54%) were castrated males. The most common breeds were crossbreeds (7, 14%), Border Collie (7, 14%), Labrador Retriever (5, 10%), and Jack Russell Terrier (4, 8%) with 24 other breeds represented (Table S1; Supplementary Material).

Clinical signs at presentation included peripheral lymphadenopathy in all dogs, lethargy (6 dogs, 12%), coughing or retching (13 dogs, 26%), polyuria and polydipsia (3 dogs, 6%), inappetence (4 dogs, 8%), weight loss (4 dogs, 8%), vomiting (3 dogs, 6%), and small intestinal diarrhoea (2 dogs, 4%). Additional abnormalities reported on physical examination, alongside peripheral lymphadenopathy, included hepatomegaly (3 dogs, 6%) and splenomegaly (4 dogs, 8%) or abdominal mass (1 dog, 2%) reported on abdominal palpation, pyrexia (rectal temperature was 39.6 °C at presentation in one dog, 3%) and heart murmur (4 dogs, 8%). Clinical substage a. (no clinical signs related to the lymphoma) was reported in 23 dogs (46%), whereas substage b. (clinical signs present due to lymphoma) was described in 27 dogs (54%) (see Appendix A.1).

All dogs had monocyte count, lymphocyte count and LMR recorded at T0, T1, T2, T3 as per inclusion criteria.

All dogs were diagnosed by cytological analysis of peripheral lymph nodes, but four dogs also had histopathology. Immunophenotyping performed in all dogs had confirmed B-cell phenotypes as follows: 7 dogs had Polymerase Chain Reaction (PCR) for antigen receptor rearrangements (PARR) performed, 4 had immunohistochemistry (IHC), 18 had immunocytochemistry (ICC) and 21 had flow cytometry (FC), being positive for markers CD21, CD79a, CD45 or PAX5.

Twenty-two dogs were staged (44%), including thoracic radiographs (13), abdominal ultrasound (21) or Computed Tomography (CT) imaging of thorax and abdomen (3). A CT scan of the thorax and abdomen alongside an abdominal ultrasound was performed on two dogs. Additional fine-needle aspirates of the liver, spleen and abdominal enlarged lymph nodes were performed in 20 dogs. Of the dogs staged, 4 were classified as stage III, 16 as stage IV and 2 as stage V (one with renal infiltration, and another dog with lung infiltration confirmed on fine-needle aspirations).

Thirty-six dogs initially received oral prednisolone alongside the chemotherapy protocol, tapered over the first 4 weeks of treatment, as per clinician discretion. All dogs had stopped prednisolone treatment by week 5.

### 3.2. Response to Treatment

Complete remission was achieved in all dogs as per the inclusion criteria. Remission was reported from week 2 (7 days), week 3 (14 days), week 4 (21 days), week 5 (28 days), week 6 (35 days), week 7 (42 days), week 8 (49 days), and week 10 (63 days) of treatment, in 5, 3, 14, 9, 8, 3, 3 and 5 dogs, respectively. Therefore, 39 dogs (78%) were reported in remission at T1 measurement (after completion of the first chemotherapy cycle (week 5 or 6 as specified depending on the chemotherapy protocol followed)), while 11 dogs (22%) were in remission at T2 measurement (week 9 or week 10 as specified depending on the chemotherapy protocol followed).

Forty-three dogs (86%) had cytology performed for confirmation of relapse. Clinical relapse in dogs without cytology (in 7 dogs) followed the previous published response evaluation criteria [18], having a 20% increase in the mean sum longest diameter of the target lymph node/s compared to baseline.

Eight dogs (16%) included in the study relapsed during the course of chemotherapy treatment, while 42 dogs (84%) completed the chemotherapy protocol. After completing chemotherapy treatment, dogs were rechecked at least every 1–2 months, or when concerned (Appendix A.2).

After confirmation of clinical relapse, 44 dogs received chemotherapy rescue protocols, including a second CEOP protocol (in 28 dogs), single-agent Lomustine—CCNU (in 15 dogs), DMAC—the cytotoxic combination of Dexamethasone, melphalan, actinomycin D, cytosine arabinoside—(in 2 dogs), single-agent epirubicin (in 2 dogs), single-agent chlorambucil (in 1 dog), while 2 dogs were prescribed palliative treatment with oral prednisolone. Two dogs did not have any additional treatments after relapse. Further patients’ details after relapse were not included in this study, as this was beyond the scope of this paper.

Dates of death, and hence survival times, were known for 25 out of 50 dogs. All dogs with known date of death, except for one, were reported to be euthanized due to lymphoma; 18 dogs were euthanized due to disease progression, one dog was suspected but not confirmed to have disease progression to involve the central nervous system due to progressive neurological signs, and four dogs were euthanized due to chemotherapy side effects during rescue chemotherapy treatment, while one dog underwent cardiac arrest, either by lymphoma disease progression or aspiration pneumonia as a complication of chemotherapy treatment. One dog was euthanized after being diagnosed with thyroid carcinoma while on rescue treatment protocol for lymphoma and progressive lethargy and hyporexia, whether due to thyroid carcinoma, lymphoma or both.

The remaining dogs were lost to follow-up but provide censored survival data (last known date alive).

### 3.3. Data Results

**Figure 2:**
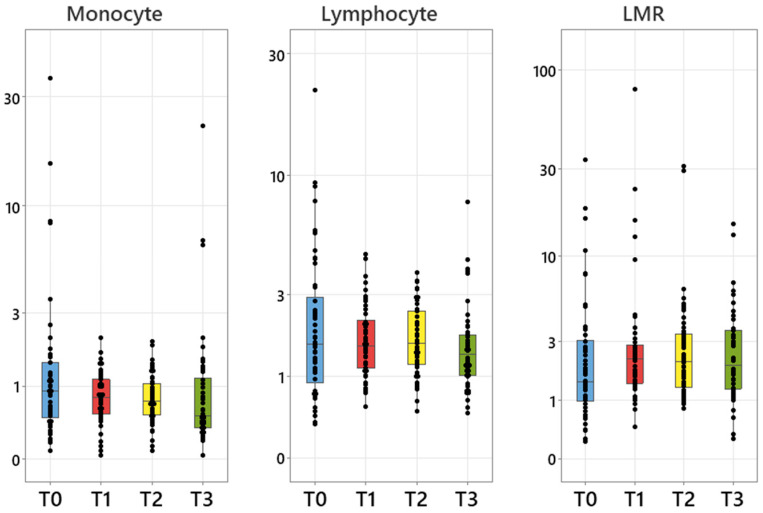
Boxplots of monocyte and lymphocyte counts (×10^9^/L) and LMR at each timepoint. Please note the use of a log scale for each graph. Solid symbols represent individual values.

Monocyte and lymphocyte counts, but not LMR, differed significantly between timepoints (Figure 2, Table S2, Supplementary Material). Both monocyte and lymphocyte count medians were lowest at T3 (relapse) with significantly lower counts than at some earlier phases (Table S2, Supplementary Material).

No significant correlations between monocyte counts, lymphocyte counts or LMR at the time of diagnosis (T0) with time to confirmed remission, disease-free interval (DFI) or overall survival (OS) were detected in this population (Table 3).

The relationships between monocyte counts, lymphocyte counts and LMR at the time of relapse (T3) and OS_r_ were analysed to determine whether they could provide additional prognostic information (Table 4). None of the T3 measures were significantly correlated with OS_r_.

**Figure 3:**
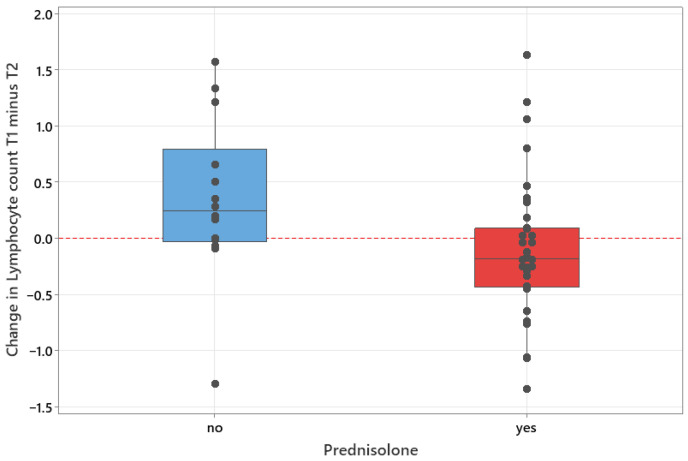
Boxplot of change in lymphocyte count between times T1 and at T2 for dogs receiving or not receiving prednisolone treatment.

Changes between T1 and T2 did not differ significantly for monocyte counts (*p* = 0.771) or LMR (*p* = 0.430) between dogs receiving or not receiving prednisolone. However, for lymphocyte counts, dogs receiving prednisolone had a median increase of 0.185 compared to those not receiving prednisolone, which had a median decrease of 0.240 (*p* = 0.007) (Figure 3).

### 3.4. Follow-Up and Median Survival Time Results

The mean follow-up for this population of dogs was 495 days (116–1995). The median disease-free interval (DFI) was 190 days (range 43–622). The median lymphoma-specific survival time for the 18 dogs that were recorded to die due to disease progression was 413 days (range 191–1995). The median overall survival time (OS) was 415 days (range 116–1995). The median overall survival from relapse (OS_r_) was 166 days (0–1658).

**Figure 4:**
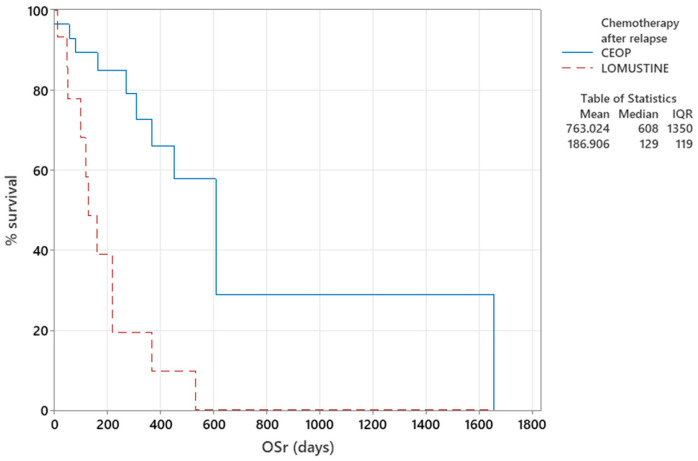
Kaplan–Meier plot of censored OS_r_ comparing group of dogs receiving CEOP-based chemotherapy (*n* = 28) as a salvage treatment, or Lomustine single-agent chemotherapy (*n* = 15).

Dogs that received another CEOP-based protocol as a salvage chemotherapy treatment survived significantly longer (*p* < 0.001) than dogs receiving Lomustine single-agent salvage protocol after first relapse (Figure 4, Table A1 in Appendix A).

**Figure 5:**
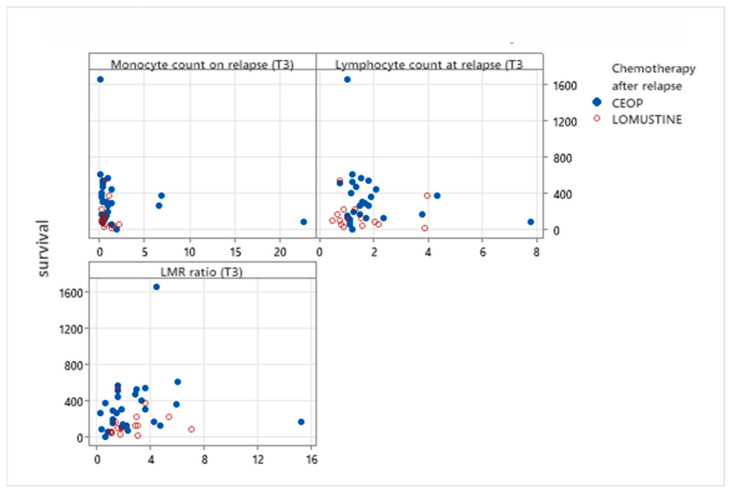
Scatterplot of OS_r_ comparing monocyte and lymphocyte counts and LMR at relapse (T3) with OS_r_ for dogs treated with salvage CEOP or Lomustine chemotherapy protocols.

The relationship between monocyte counts, lymphocyte counts and LMR at the time of relapse (T3) with OS_r_ for the dogs receiving either CEOP protocol or single-agent Lomustine as salvage chemotherapy after first relapse is shown in Table 5 and Figure 5. The only significant correlation was between monocyte count at T3 and OS_r_ in the CEOP group.

## 4. Discussion

In this study, we evaluated the monocyte and lymphocyte counts alongside the LMR in dogs diagnosed with DLBCL across different timepoints during the course of their chemotherapy treatment and at relapse to assess the variation in the parameters and their prognostic value in our population of dogs.

Our main objective was to evaluate the monocyte counts, lymphocyte counts, and the LMR at the time of relapse in dogs with DLBCL.

Given that LMR was previously shown to be a marker for shorter TTP, shorter OS and an independent prognostic indicator in dogs with multicentric lymphoma [8,20,21,28], and that the absolute monocyte count was independently associated with time to progression (TTP) and lymphoma-specific survival (LSS) [21], we first sought to evaluate this in our population of dogs as well. Absolute monocyte and lymphocyte counts at diagnosis were not found to be significantly associated with DFI and OS in this population of dogs. Statistically significant association with absolute lymphocyte count at the time of diagnosis has equally not been shown in any of the previous studies. Regarding the absolute monocyte count, this is in agreement with the study of Mutz et al. [22]. In contrast, it diverges from the study of Marconato et al. where absolute monocyte count at diagnosis was associated with TTP [21] and the study of Perry et al. [19] that found that greater monocyte count was related to lower DFI. Furthermore, no statistically significant association between LMR at the time of diagnosis and the time to achieve remission, DFI or OS was found in our population of dogs, which concurs with the study of Henriques et al. [20], where association between LMR and lymphoma progression and survival was not found, but differs from other studies that found significant association [8,20,21,28]. Previous studies that found significant association included dogs that were completely staged [21,28], different sample size, and variation in chemotherapy protocols compared to our population, all of which could have influenced the results.

Absolute lymphocyte count at the time of relapse was not found to be statistically significantly associated with OS_r_ in this population of dogs. Interestingly, monocyte count at relapse was found to be statistically correlated with OS_r_ solely in the group of dogs treated with CEOP salvage protocol after first relapse in our study. However, this finding was not detected for the overall population of dogs treated with any other chemotherapy rescue protocol, albeit the latter was a much smaller sample. This finding suggests that monocyte count at relapse in dogs could be used as a potential surrogate marker of the host immunity at relapse, and therefore, as a prognostic factor, but the interpretation of this finding must be taken with caution given the small size of the group assessed and failing to observe the same result in the overall population. No equivalent results have been found in dogs; however, absolute lymphocyte count at relapse was found to be an independent prognostic factor for PFS and OS in people with diffuse large-cell lymphoma [39]. This encourages the continued studying of the validity of monocyte and lymphocyte counts as prognostic markers at time of relapse, which still needs to undergo further investigation in a larger population of dogs.

LMR at the time of relapse was also not found to be statistically significant associated for OS_r_. Our findings differs from human studies, where LMR < 2.6 at relapse was significantly associated with shorter overall survival time compared to the higher LMR group [38], or in the study by Li et al. [24] where LMR < 2.0 at the time of first relapse was found to be a biomarker combining an estimate of host immune homeostasis and tumour microenvironment, and an adverse prognostic factor for overall survival [24]. The difference between these studies and our results may be related to the smaller population of dogs in our study which could have led to type II error, the difference in chemotherapy protocols between humans and dogs, difference in bone marrow recovery dynamics, or long-term monocyte or lymphocyte behaviour. Although there are strong similarities in blood-cell-based biomarkers between humans and dogs [40], differences have also been reported, such as stronger monocyte IFN-γ responsiveness or lower lymphocyte cytokine output in dogs [41], which could differ in blood cell results between both. Moreover, not all the dogs included in our study had their time of death recorded, and the time of last contact was included as censored data, which may have affected the final statistics.

Additionally, the monocyte and lymphocyte counts, alongside the LMR, were evaluated at different timepoints during the course of their first-line chemotherapy treatment and at relapse. The absolute lymphocyte count was found to be lowest at the time of relapse in our population of dogs, consistent with lymphocyte depletion associated with tumour physiology during tumour progression [42,43]. Lymphocytes mediate humoral and cellular antitumour immune responses, with depression of absolute numbers of T-cells due to the rapid lymphocyte turnover and spontaneous apoptosis of circulating CD8+ antigen-responding effector T-cells, showing the host immunological incompetence during cancer development [42]. Lymphopenia is also associated with poor prognosis in high-grade lymphomas in people [43]. This is the first study to find statistically significant differences in lymphocyte count at the time of relapse when compared to other timepoints in dogs with diffuse large-cell lymphoma receiving chemotherapy, suggesting that monitoring lymphocyte counts in these dogs could have clinical importance, since lower counts at relapse when compared to remission may indicate disease progression and pending clinical relapse. Further prospective studies are needed to better understand the role of lymphopenia in canine tumour relapse and to assess if lymphocyte count could be used as an independent biomarker of relapse in lymphoma patients.

Interestingly, the absolute monocyte count was also lowest at relapse, contrasting with expectations based on tumour physiology [44,45,46], in which a higher monocyte count at relapse, similar to that observed at diagnosis [19], would typically be expected. Monocytosis is correlated with a poor prognosis in people with cancer [47] and as an adverse prognostic factor in multiple solid tumours [48], given that monocytes contribute to the suppression of host antitumour immunity supporting the survival and proliferation of neoplastic B-cells and supressing the proliferation of normal T cells, and promoting tumour cell extravasation, angiogenesis, contributing to tumour growth [44,47]. One possible explanation for our finding is that monocyte dynamics may differ during tumour progression compared to initial diagnosis, though data on monocyte trends at relapse in dogs are lacking. It has been hypothesised that the pronounced monocyte response seen at tumour onset could be reduced at relapse in dogs previously treated with chemotherapy. Monocyte recruitment into tissues during relapse, or bone marrow infiltration could be other explanations for this finding. However, there is no clear support for this result and further studies including immunophenotyping (for surface markers) or in combination with other biomarkers with cytokines are needed to evaluate this finding further. Similarly, questions regarding monocyte subsets and their changes at relapse remain open in human medicine [24].

LMR was also not statistically significant different at relapse in this study population when compared to other timepoints, which differs from studies in people, where LMR at follow-up was concluded to be a biomarker for relapse after treatment [45].

It is important to consider that chemotherapy-induced myelosuppression may have directly reduced monocyte and lymphocyte counts, potentially confounding their use as immune biomarkers and having a direct impact on the LMR results. Suppression of lymphocyte regeneration following chemotherapy in dogs treated for lymphoma have been postulated [46], although prior studies have not demonstrated a significant effect of chemotherapy on monocyte counts [49]. Chemotherapy impact on cell counts at relapse following chemotherapy treatment and their use as immune biomarkers has not been explicitly discussed in previous human studies [24,38]. However, blood samples were collected at least 14 days after the last chemotherapy dose at T1 and T2 and this interval was thought to minimise potential treatment-related cytopenias. However, at T3, a subset of dogs had relapsed while still receiving chemotherapy, and therefore, the influence of prior treatment on their blood cell counts cannot be entirely excluded.

No statistical significance was found in monocyte or lymphocyte counts between T1 or T2, and the use of prednisolone was thought to not affect our results. The significant difference in changes between lymphocyte count (*p* = 0.007) increased at T2 in dogs receiving prednisolone, which is thought to be related to the ceasing of prednisolone in some dogs before T2, with its subsequent rise due to glucocorticoid-induced apoptosis and redistribution to the lymphoid tissue of lymphocytes [50].

Finally, we observed that dogs treated with CEOP-based rescue chemotherapy protocol, compared to the group treated with Lomustine-based single-agent chemotherapy had significantly improved survival times (*p* < 0.001 for log-rank test), in accordance with a previous study [51], and confirming improved prognosis in dogs treated with multiagent protocols. However, dogs that achieved longer DFI (>214 days in 93% of the 28 dogs) were more likely to receive CEOP rescue chemotherapy protocol, compared to dogs with shorter DFI (<196 days for 70% of the 15 dogs) who were more likely to receive single-agent Lomustine rescue chemotherapy protocol. This may have biassed the final results.

There are limitations in this study due to its retrospective nature and relatively small sample size; the latter may have limited statistical power and possibly led to missing important associations (type II errors).

One important limitation is the use of different blood cell count machines; two main external laboratories were used for haematology results, with differences in the reference range.

Not all dogs were staged at diagnosis, and the dogs were not restaged at regular intervals following completion of the protocol, which means they could have relapsed prior to identification of peripheral lymphadenopathy. This limitation could have affected results. A larger sample with complete data would be needed to completely exclude this hypothesis.

All dogs included were confirmed to have B-cell type diffuse lymphoma either by ICC, ICH, FC or PARR. Based on previous veterinary literature, antibodies against lymphocyte markers on tissue biopsy or cytology specimens (IHC or ICC, respectively), or on individual cells in a fluid medium (FC) should be used for accurate determination and reliability of immunophenotyping [52]. However, PARR should be mainly used to confirm clonality in a population of lymphoid cells [53] and not immunophenotyping, given that the agreement between PARR and IHC has been reported at just 69%, and between PARR and FC at 63% [54].

The treatment protocol was also not completely standardised (including dogs with CEOP and modified-CEOP protocol). Nevertheless, the main objective of our study was to assess the blood cell count differences between response and relapse to treatment, and given that all dogs received the same drugs during the course of chemotherapy, and responded to treatment and relapsed afterwards (following precise response evaluation criteria [18] and cytological examination), it is less likely suggested that a lack of further treatment standardisation would have substantially biassed the results.

## 5. Conclusions

Our study did not identify statistical significance association between absolute monocyte, lymphocyte counts or LMR with OS_r_ at the time of relapse for our population of dogs with multicentric lymphoma.

Larger, controlled prospective studies evaluating absolute monocyte and lymphocyte counts throughout the course of chemotherapy and at relapse in a larger population of dogs are needed to assess our findings further and assess whether these counts have clinical utility in detecting disease progression.

## Figures and Tables

**Table 1:** Multidrug CEOP chemotherapy protocol (UW–Madison–Short) used in 33 dogs.

DRUG	WEEK																	
	1	2	3	4	5	6	7	8	9	10	11	13	15	17	19	21	23	25
Vincristine IV	X		X			X		X			X		X		X		X	
Cyclophosphamide PO		X					X					X				X		
Epirubicin IV				X					X					X				X
Prednisolone PO	X	X	X	X														

“X” denotes the specific week in which the drug was administered during the chemotherapy course. IV (intravenous); PO (oral). Vincristine dose (0.5–0.75 mg/m^2^) IV; cyclophosphamide dose (200–250 mg/m^2^) PO; epirubicin dose (30 mg/m^2^) IV.

**Table 2:** Modified—CEOP chemotherapy protocol used in the remaining 17 dogs.

DRUG	WEEK															
	1	2	3	4	5	6	7	8	9	10	11	12	13	14	15	16
Vincristine IV	X				X				X				X			
Cyclophosphamide PO		X				X				X				X		
Epirubicin IV			X				X				X				X	
Prednisolone PO	X	X	X	X												

“X” denotes the specific week in which the drug was administered during the chemotherapy course. IV (intravenous); PO (oral). Vincristine dose (0.5–0.75 mg/m^2^) IV; cyclophosphamide dose (200–250 mg/m^2^) PO; epirubicin dose (30 mg/m^2^) IV.

**Table 3:** Spearman rank correlations between monocyte and lymphocyte counts and LMR at diagnosis (T0) and the day of first documented remission, disease-free interval—DFI and overall survival—OS (including censored data).

	1st Remission		DFI		OS	
	r_s_	*p*	r_s_	*p*	r_s_	*p*
Monocyte count T0	0.122	0.399	0.151	0.294	0.092	0.524
Lymphocyte count T0	−0.032	0.826	−0.036	0.801	−0.008	0.956
LMR T0	−0.177	0.218	−0.159	0.270	−0.176	0.222

LMR (lymphocyte-to-monocyte ratio), DFI (disease-free interval), OS (overall survival time).

**Table 4:** Spearman rank correlations (r_s_) between monocyte count, lymphocyte count and LMR at the time of relapse (T3) and OS_r_ (overall survival at relapse, including censored data).

	OS_r_ (Overall Survival from Relapse)	
	r_s_	*p*
Monocyte count T3	−0.209	0.146
Lymphocyte count T3	−0.012	0.932
LMR T3	0.136	0.347

OS_r_ (overall survival time); LMR (lymphocyte-to-monocyte ratio).

**Table 5:** Spearman rank correlations (rs) between monocyte, lymphocyte and LMR at relapse (T3) with OS_r_ for dogs treated with salvage CEOP rescue protocols (*n* = 28) or treated with salvage LOMUSTINE protocol (*n* = 15).

	OS_r_			OS_r_	
CEOP Group	r_s_	*p*	LOMUSTINE Group	r_s_	*p*
Monocyte count T3	−0.419	0.026	Monocyte count T3	−0.484	0.067
Lymphocyte count T3	−0.013	0.949	Lymphocyte count T3	−0.266	0.337
LMR T3	0.346	0.071	LMR T3	0.336	0.221

OS_r_ (overall survival time); LMR (lymphocyte-to-monocyte ratio).

## Data Availability

Data are available at https://docs.google.com/spreadsheets/d/1RhngvIftPcMIGMRv4YAsccXVGscwgpPC/edit?usp=sharing&ouid=117312908401678382372&rtpof=true&sd=true (accessed on 15 December 2025).

## Supplementary Materials

The following supporting information can be downloaded at https://www.mdpi.com/article/10.3390/ani16010009/s1, Figure S1: Diagram of exclusion criteria; Table S1: Dog breeds included in the study; Table S2: Median (range) of monocyte and lymphocyte counts (×10^9^/L) and LMR at four different timepoints of chemotherapy treatment.

## Abbreviations

The following abbreviations are used in this manuscript:

LMR	Lymphocyte-to-monocyte ratio
CHOP protocol	A multi-agent cytotoxic chemotherapy protocol used in the treatment of canine lymphoma that uses 4 different drugs—Vincristine, Cyclophosphamide, Doxorubicin and Prednisolone
CEOP protocol	CEOP is a multi-agent cytotoxic chemotherapy protocol used in the treatment of canine lymphoma that used 4 different drugs—Vincristine, Cyclophosphamide, Epirubicin and Prednisolone
OS	Overall survival
OS_r_	Overall survival from relapse
TTP	Time to progression
PARR	Polymerase Chain Reaction (PCR) for antigen receptor rearrangements
IHC	Immunohistochemistry
ICC	Immunocytochemistry
FC	Flow cytometry
RR	Remission rate
DFI	Disease-free interval
MST	Median survival time
ORR	Overall response rate
DLBCL	Diffuse large B-cell lymphoma
DL	Diffuse lymphoma
LSS	Lymphoma-specific survival
PFR	Progression-free survival

## Appendix A

### Appendix A.1

Neutropenia was defined when the absolute neutrophil count was below the reference range for the analyser (<2.8–2.95 × 10^9^/L); neutrophilia was reported if the absolute neutrophil count was above the reference range for the analyser (>11.64–13.6 × 10^9^/L); anaemia was reported if the haematocrit was below the reference range for the analyser (<37–37.3%), and defined as non-regenerative if the reticulocyte count was < 90–110 × 10^9^/L.

Laboratory abnormalities reported at initial presentation included neutrophilia (6, 12%), elevation of ALKP (13, 26%) and ALT (10, 20%) liver enzyme activity, non-regenerative anaemia (6, 12%), azotaemia (3, 6%), and atypical lymphocytes observed on blood smear (5, 10%).

### Appendix A.2

Variations in chemotherapy treatment were as follows: in five dogs, the first cycle of the CEOP protocol was altered, receiving cyclophosphamide in week 1, followed by vincristine in week 2, due to pending results of MDR1 genetic mutation testing. All dogs with MDR1 results (5) were negative, and the rest of the protocol was unaltered. Chlorambucil (20 mg/m^2^ PO) was used instead of cyclophosphamide due to the development of urinary tract infection in one patient from week 7/25. Mitoxantrone (6.5 mg/m^2^ IV) was used instead of epirubicin in two dogs given the presence of an underlying cardiac disease [1 dog] (with reported decreased fractional shortening on echocardiography), or due to developing grade 3 diarrhoea alongside grade 2 anorexia [55] after epirubicin injection in another dog. Three dogs received intravenous vinblastine (2.5 mg/m^2^) instead of vincristine after one dog developed grade 2 diarrhoea, grade 3 enteritis and vomiting [55]. Five dogs had a 10% dose reduction in vincristine due to the development of grade 2 neutropenia (1), grade 4 neutropaenia [56] (1) or grade 3 diarrhoea [55] (in 1 dog), and grade 1 anorexia [55] (in 1 dog). In one patient. the dose of cyclophosphamide was reduced at 10% due to grade 2 diarrhoea and grade 2 neutropenia. A 5% reduction in epirubicin was also made in 5 dogs due to grade 2 (in 2 dogs) and grade 3 diarrhoea (in 3 dogs). Chemotherapy was delayed (4–7 days) in 13 dogs at least once due to developing neutropenia. In 3 dogs, L-asparaginase was given at the initiation of treatment at 10.000 IU/m^2^.

### Appendix A.3

**Table A1:** Comparison of OS_r_ between two groups: group receiving CEOP chemotherapy (*n* = 28) and group receiving a single-agent Lomustine (*n* = 15) as a salvage chemotherapy treatment after the first relapse.

	CEOP		Lomustine		*p*
	Median	Range	Median	Range	
OS_r_ (days)	284	0–1658	99	10–532	0.005
