# Supplementary material for: Monocyte and Lymphocyte Count, and Lymphocyte/Monocyte Ratio as Prognostic Factors at the Time of First Relapse in Canine Diffuse Large B-Cell Lymphoma Patients Receiving Chemotherapy
Source: Animals (Basel). 2025 Dec 19;16(1):9. doi: 10.3390/ani16010009 (PMC12784993; doi:10.3390/ani16010009)
Supplement: Supplementary file 1 [file animals-16-00009-s001.zip › animals-3981344-supplementary.pdf]

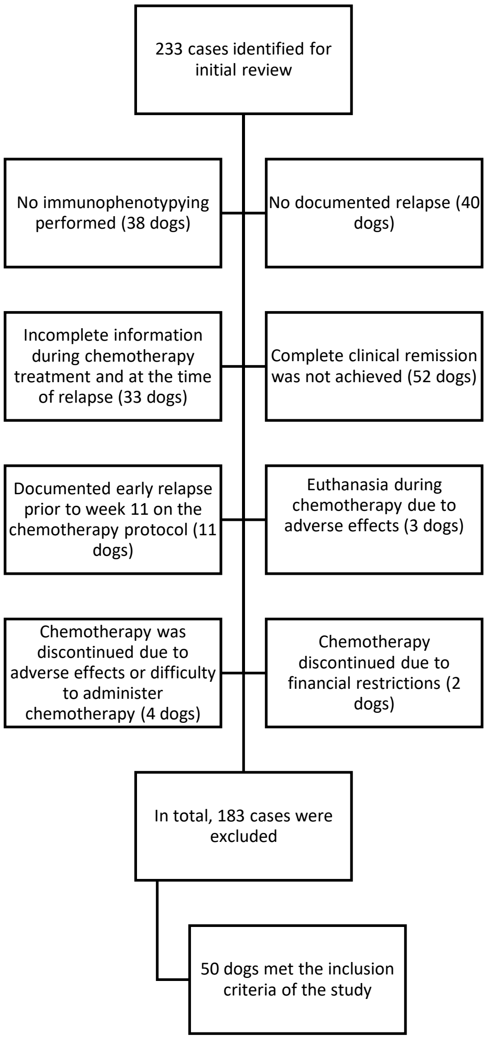


**Figure S1.** Diagram of exclusion criteria.

**Table S1.** Dog breeds included in the study.

| Breed | n | % |
|---|---|---|
| Australian Kelpie | 1 | 2 |
| Australian Labradoodle | 1 | 2 |
| Beagle | 1 | 2 |
| Bernese Mountain | 1 | 2 |
| Border Collie | 7 | 14 |
| Boxer | 1 | 2 |
| Bull Terrier | 1 | 2 |
| Bulldog | 1 | 2 |
| Cairn Terrier | 1 | 2 |
| Cavapoo | 1 | 2 |
| Cockapoo | 1 | 2 |
| Cocker Spaniel | 1 | 2 |
| Collie | 1 | 2 |
| Crossbreed | 7 | 14 |
| French Bulldog | 2 | 4 |
| German Pointer | 1 | 2 |
| Golden Retriever | 1 | 2 |
| Hungarian Vizsla | 1 | 2 |
| Irish Terrier | 1 | 2 |
| Jack Russell Terrier | 4 | 8 |
| Labradoodle | 1 | 2 |
| Labrador Retriever | 5 | 10 |
| Shar-pei | 1 | 2 |
| Springer Spaniel | 1 | 2 |
| Staffordshire Bull Terrier | 2 | 4 |
| Tibetan Terrier | 2 | 4 |
| West Highland White Terrier | 1 | 2 |
| Yorkshire Terrier | 1 | 2 |

**Table S2.** Median (range) of monocyte and lymphocyte counts (x109/L) and LMR at four different timepoints of chemotherapy treatment. Data were analyzed using Friedman tests followed by pairwise comparisons where significant. Medians within a column not sharing letters in common were significantly different.

| | | ***Monocyte count*** | ***Lymphocyte count*** | ***LMR*** |
|---|---|---|---|---|
| *T0* | **(at diagnosis)** | $0.91^{b}$ (0.09-35.88) | $1.62^{ab}$ (0.33-21.76) | $1.50^{a}$ (0.23-33.71) |
| *T1* | **(after completion of 1rst chemo cycle)** | $0.80^{ab}$ (0.04-2.16) | $1.60^{ab}$ (0.55-4.62) | $2.25^{a}$ (0.46-79.25) |
| *T2* | **(after completion of 2nd chemo cycle)** | $0.74^{ab}$ (0.09-2.06) | $1.64^{b}$ (0.49-3.82) | $2.18^{a}$ (0.82-31.22) |
| *T3* | **(relapse)** | $0.50^{a}$ (0.04-22.57) | $1.41^{a}$ (0.47-7.79) | $2.04^{a}$ (0.28-15.20) |
| | **p** | 0.009 | 0.026 | 0.093 |

*LMR (lymphocyte-to-monocyte ratio).
